# Establishment of a Site-Specific Tropospheric Model Based on Ground Meteorological Parameters over the China Region

**DOI:** 10.3390/s17081722

**Published:** 2017-07-27

**Authors:** Chongchong Zhou, Bibo Peng, Wei Li, Shiming Zhong, Jikun Ou, Runjing Chen, Xinglong Zhao

**Affiliations:** 1 State Key Laboratory of Geodesy and Earth’s Dynamics, Institute of Geodesy and Geophysics, Chinese Academy of Sciences, No. 340 Xudong Road, Wuhan 430077, China; bobby@asch.whigg.ac.cn (B.P.); liwei@asch.whigg.ac.cn (W.L.); smzhong@asch.whigg.ac.cn (S.Z.); ojk1009@public.wh.hb.cn (J.O.); zhaoxinglong15@mails.ucas.ac.cn (X.Z.); 2 College of Earth Sciences, University of Chinese Academy of Sciences, No. 19A Yuquan Road, Beijing 100049, China; 3 School of Computer and Information Engineering, Xiamen Institute of Technology, No. 600 Ligong Road, Jimei District, Xiamen 361024, China; chenrj@xmut.edu.cn

**Keywords:** global tropospheric model, single-site tropospheric model, radiosonde data, ZTD, SAAS_S model, CH_S model

## Abstract

China is a country of vast territory with complicated geographical environment and climate conditions. With the rapid progress of the Chinese BeiDou satellite navigation system (BDS); more accurate tropospheric models must be applied to improve the accuracy of navigation and positioning. Based on the formula of the Saastamoinen and Callahan models; this study develops two single-site tropospheric models (named SAAS_S and CH_S models) for the Chinese region using radiosonde data from 2005 to 2012. We assess the two single-site tropospheric models with radiosonde data for 2013 and zenith tropospheric delay (ZTD) data from four International GNSS Service (IGS) stations and compare them to the results of the Saastamoinen and Callahan models. The experimental results show that: the mean accuracy of the SAAS_S model (bias: 0.19 cm; RMS: 3.19 cm) at all radiosonde stations is superior to those of the Saastamoinen (bias: 0.62 cm; RMS: 3.62 cm) and CH_S (bias: −0.05 cm; RMS: 3.38 cm) models. In most Chinese regions; the RMS values of the SAAS_S and CH_S models are about 0.51~2.12 cm smaller than those of their corresponding source models. The SAAS_S model exhibits a clear improvement in the accuracy over the Saastamoinen model in low latitude regions. When the SAAS_S model is replaced by the SAAS model in the positioning of GNSS; the mean accuracy of vertical direction in the China region can be improved by 1.12~1.55 cm and the accuracy of vertical direction in low latitude areas can be improved by 1.33~7.63 cm. The residuals of the SAAS_S model are closer to a normal distribution compared to those of the Saastamoinen model. Single-site tropospheric models based on the short period of the most recent data (for example 2 years) can also achieve a satisfactory accuracy. The average performance of the SAAS_S model (bias: 0.83 cm; RMS: 3.24 cm) at four IGS stations is superior to that of the Saastamoinen (bias: −0.86 cm; RMS: 3.59 cm) and CH_S (bias: 0.45 cm; RMS: 3.38 cm) models.

## 1. Introduction

In navigation and positioning applications of the Global Navigation Satellite System (GNSS), one of the main error sources is the atmospheric delay error. This delay error is divided into ionospheric delay and tropospheric delay [1]. Since the effect of ionospheric delay can be mostly eliminated using dual-frequency observation technology, the tropospheric delay remains one of the main error sources for navigation and positioning of GNSS data. The amount of zenith tropospheric delay is about 2.3 m, but the delay can reach 20 m when the elevation angle is below 10 degrees [2,3]; therefore, elimination of the delay is required in high-accuracy navigation and positioning. Commonly, the slant tropospheric delay is obtained by projecting the zenith delay into the slant direction with a mapping function. Many ZTD models have been developed to reduce the signal influence caused by the troposphere. ZTD can be divided into two parts: the Zenith Hydrostatic Delay (ZHD) and the Zenith Wet Delay (ZWD). Presently, China is progressively developing the Chinese BeiDou Navigation Satellite System; therefore, it is of great importance to provide high-accuracy ZTD correction services for massive BDS/GNSS usage over China.

Based on the formulas given by Smith and Weintraub [4], the calculation of the ZHD and ZWD is closely related to meteorological parameters at the observation station. Therefore, many researchers put forward tropospheric delay models based on pressure, temperature, and water vapor pressure at the surface level, such as the Hopfield model [5], the Saastamoinen model [6], the Black model [7], etc. These tropospheric models are established based on the atmospheric troposphere delay theory, and the model coefficients are usually uniform all over the globe. Generally, the ZHD can be accurately determined with the Saastamoinen model, but the accuracy of the estimated ZWD is relatively low. Therefore, new formulas for ZWD estimation have been developed, resulting in new models, such as the Berman model [8], the Callahan model [9], and the Ifadis model [10].

Additionally, in order to accommodate the applications for real time positioning and kinematic navigation, a series of tropospheric models which are not dependent on observed meteorological parameters have been developed. For example, the UNB models (UNB1 through UNB4) and the EGNOS model [11,12,13,14]. These two models can only account for latitudinal variation, so the models commonly result in large biases in some regions [15]. In recent years, many multi-dimensional grid tropospheric delay models for regional or global applications have been developed, such as the Global ZTD (GZTD) model [16], the empirical global ZTD model IGGtrop [15], the ZTD model developed by Zhao et al. [17], and the GPT model and its updated version GPT2w model developed by Boehm et al. [18,19].

The above two types of models were developed for global or regional applications, and their accuracies are usually at the few cm level; however, the model accuracies commonly obviously decrease in some areas with complex weather change [20]. With five years of GNSS-ZTD data from 25 stations of the Crustal Movement Observation Network of China (CMONOC), Zhang et al. showed that the mean Root Mean Square (RMS) value of the IGGtrop model for China was 4.40 cm, but there were large biases at some stations [21]. Using radiosonde data for 2003–2012 from 92 stations over China, Chen et al. [22] found that the mean accuracies of ZHD and ZWD estimation values for the Saastamoinen model were 0.84 cm and 3.82 cm respectively and that the mean accuracies of the Callahan and Ifadis models were 3.90 cm and 4.02 cm respectively for computing the ZWD value. Although those three models based on surface meteorological parameters show better performance than the blind model—IGGtrop, they show larger biases in both ZHD and ZWD in the low latitude regions compared to results in the higher latitudes, and their accuracies are relatively lower in the low latitude regions than higher latitudes [22]. China is a very large country, and the geographical environment and climate are very complex [15,21]; therefore, it is very hard to insure that these tropospheric models with uniform coefficients have a consistent accuracy for all the regions in China. In addition, through our analysis, we have found that the coefficients of the Saastamoinen model may have dramatic deviation from the real local tropospheric environment. To overcome these problems, this study attempts to establish a tropospheric model for specific regions and specific environments in China. Based on the formula of the Saastamoinen and Callahan models, this paper develops two single-site tropospheric models for China using radiosonde data from 2005 to 2012. In addition, we assess the two single-site tropospheric models and the Saastamoinen, Callahan, and GPT2w models using radiosonde data for 2013 and ZTD data from four International GNSS Service (IGS) stations.

This paper is structured as follows: Section 2 describes the basic theory and models for the tropospheric delay while data presentation and methodology are shown in Section 3. Section 4 presents the experimental results for each models obtained with two kinds of data and the spatial distributions of the SAAS_S model coefficients. The conclusions are given in Section 5.

## 2. Basic Theory and Models for Tropospheric Delay

### 2.1. Tropospheric Delay

In satellite navigation and positioning, tropospheric delay occurs in the process of the electromagnetic wave signal passing through the unionized neutral atmosphere, the height range of which is between the surface and 50 km. The delay in zenith direction generated by the radio signal can be obtained with Equation (1):

(1) $$\mathrm{ZTD}=10^{-6}\int_{h_{0}}^{\infty }N\cdot dh$$

where N is the atmospheric refractivity (unitless), and $h_{0}$ is the height of the observation station above mean sea level (unit: m).

According to Equation (1), the ZTD can be obtained by computing the integral for the atmospheric refractivity at different heights. With the empirical equation given by Smith and Weintraub, the atmospheric refractivity can be expressed by the following Equation [23]:

(2) $$N=k_{1}\frac{\left(P-e\right)}{T}+k_{2}\frac{e}{T}+k_{3}\frac{e}{T^{2}}$$

where $k_{1}$, $k_{2}$, and $k_{3}$ are the refractivity constants, with values of 77.6890 K/hPa, 71.2952 K/hPa, and 375,463 K^2^/hPa respectively. T stands for the temperature (unit: kelvin), P and e are the total pressure and the water vapor pressure respectively (unit: hectopascal). N consists of two parts, one is $N_{h}$, which refers to the dry component of atmospheric refractivity, the other is $N_{w}$, which is the wet component of atmospheric refractivity. $N_{h}$ and $N_{w}$ can be calculated with Equations (3) and (4) as follows:

(3) $$N_{h}=k_{1}R_{d}\rho$$

(4) $$N_{w}=k_{2}^{\prime }\cdot \frac{e}{T}+k_{3}\frac{e}{T^{2}}$$

(5) $$k_{2}^{\prime }=k_{2}-k_{1}\frac{R_{d}}{R_{w}}$$

where, $R_{d}=287.053\;J\cdot K^{-1}\cdot kg^{-1}$ and $R_{w}=461.495\;J\cdot K^{-1}\cdot kg^{-1}$ are the gas constants for dry air and water vapor, respectively; ρ is the air density.

Substituting Equations (4) and (5) into Equation (1), we obtain:

(6) $$\mathrm{ZTD}=10^{-6}\int_{h_{0}}^{\infty }N_{h}\cdot dh+10^{-6}\int_{h_{0}}^{\infty }N_{w}\cdot dh$$

The first term on the right-hand side of Equation (6) is the ZHD and the last term is the ZWD. Several studies have shown that the calculation of the hydrostatic delay is theoretically derivable based on the assumption that air is an ideal gas and that the troposphere satisfies the hydrostatic equilibrium [24,25,26]. Therefore, the ZHD can be calculated by:

(7) $$\mathrm{ZHD}=10^{-6}k_{1}R_{d}\frac{P_{0}}{g_{m}}$$

where $g_{m}$ is the gravity acceleration at the centroid of the vertical atmosphere column (unit: meters per square second). In fact, Equation (7) also represents the Saastamoinen ZHD model [6].

From the above analyses, ZTD is divided into two parts, the ZHD and the ZWD. There are two methods to acquire the ZWD, one is directly computing the integral for $N_{w}$ [24], the other is computing the difference between the ZTD and the computed ZHD. This paper adopts the latter method to calculate the ZWD.

### 2.2. Tropospheric Models

This paper principally researches and analyzes the experiment results for six tropospheric models over China. It should be noted that the Saastamoinen, GPT2w, and Callahan models use the original formulas and coefficients, and the GPT2w_S model is the GPT2w model based on surface observation meteorological parameters. In addition, the SAAS_S and CH_S models are respectively the single-site model of Saastamoinen and Callahan models. Detailed information about these models are provided in below sections.

#### 2.2.1. Saastamoinen Model

Among the tropospheric models based on measured meteorological parameters, the Saastamoinen model [6] is a high-accuracy ZTD model with wide applications. The equation for the model reads:

(8) $$\mathrm{ZTD}=0.002277\times \frac{\left[P_{0}+\left(0.05+\frac{1255}{T_{0}}\right)e_{0}\right]}{f\left(\phi ,h\right)}$$

(9) f(ϕ,h)=1−0.00266cos2ϕ−0.00028 h

where $P_{0}$, $T_{0}$, and $e_{0}$ are respectively the surface pressure (unit: hectopascal), the surface temperature (unit: kelvin), and the water vapor pressure at the surface level (unit: hectopascal), and *ϕ* stands for station latitude (unit: radians). From Equations (8) and (9), the ZTD can be obtained by importing the surface pressure, the surface temperature, the water vapor pressure at the surface level, and the station’s latitude and the height above mean sea level. The Saastamoinen model will be referred to as the SAAS model in this article.

#### 2.2.2. GPT2w Model

The global pressure and temperature 2 wet (GPT2w) model developed by Boehm et al. is an improved troposphere delay model for global pressure and temperature (GPT), which provides mean values plus annual and semiannual amplitudes of pressure, temperature and its lapse rate, water vapor pressure and its decrease factor, weighted mean temperature, as well as hydrostatic and wet-mapping function coefficients of the Vienna mapping Equation (1) [19]. The value of the ZWD in the GPT2w model is computed using Equations (10) and (11), and the ZHD is obtained with Equation (7).

(10) $$\mathrm{ZWD}=10^{-6}\times \left(k_{2}^{\prime }+\frac{k_{3}}{T_{m}}\right)\times \frac{R_{d}\times e_{0}}{\left(\lambda +1\right)g_{m}}$$

(11) $$T_{m}=T_{0}\times \left(1-\frac{\alpha \times R_{d}}{\left(\lambda +1\right)g_{m}}\right)$$

where $k_{2}^{\prime }$, $k_{3}$, and $R_{d}$ have been defined in Equations (2)–(5), $T_{0}$, $e_{0}$, *ϕ*, and h have been defined in Equation (9), λ is the water vapor decrease factor (unitless), and α refers to the temperature lapse rate (unit: kelvin per kilometer). In fact, the GPT2w model meshes the atmospheric parameters temporally and spatially; therefore, GPT2w also represents a type of gridded single-site model. By importing longitude, latitude, and altitude for the station, as well as DoY (Day of Year), we can use a parameter list for the GPT2w model to compute surface pressure, surface temperature, water vapor pressure at the surface level, lapse rate of temperature, and attenuation factor of water vapor pressure at the station, and the ZTD can be obtained by integrating Equations (7), (10) and (11). In addition, the ZTD can be calculated not only using the above parameter table acquired by the GPT2w model but also by using measured meteorological parameters. Therefore, we will determine the accuracy of the above-mentioned two methods for computing the ZTD and compare and analyze the differences between the above mentioned two methods and the other tropospheric models. These two methods are called GPT2w and GPT2w_S respectively in this paper.

#### 2.2.3. Callahan Model

The ZWD model developed by Callahan assumes that the water vapor pressure distribution is empirically expressed by the following equation [9]:

(12) $$e=e_{0}\cdot \exp\left(-2.88\times 10^{-4}\cdot h-4.8\times 10^{-8}\cdot h^{2}\right)$$

Based on this assumption, Callahan derived the ZWD model using water vapor pressure and temperature [9]. This model is called the CH model in this paper and the calculation of the model is given in Equation (13):

(13) $$\mathrm{ZWD}=a_{1}\frac{e_{0}}{T_{0}^{2}}$$

where $a_{1}$ is set as 1035 (unit: $m\cdot K^{2}/\mathrm{hPa}$). In addition this study develops a single-site tropospheric model for each observation station based on the Callahan formula and recalculates $a_{1}$ at each station using a large data volume. The single-site model for Callahan is called CH_S in this paper.

#### 2.2.4. SAAS_S Model

According to the formula of the Saastamoinen model, this paper establishes a single-site tropospheric model based on pressure, temperature, and water vapor pressure at the surface level. The formula of the single-site model is given in Equation (14) and this model is called SAAS_S. In order to absorb residual errors, the equation has one additional constant term compared to the Saastamoinen model. This study will analyze further the distribution of the coefficients of the SAAS_S model.

(14) $$\mathrm{ZTD}=a_{1}P_{0}+a_{2}\frac{e_{0}}{T_{0}}+a_{3}e_{0}+a_{4}$$

where $a_{1}$, $a_{2}$, $a_{3}$, and $a_{4}$ are the coefficient values of the model and their units are m/hPa, m⋅K/hPa, m/hPa, and m respectively. In this paper, we aim to work out the model coefficients for each station using a large number of data.

Based on the above details, the SAAS, SAAS_S, GPT2w, and GPT2w_S models are ZTD models, but the CH and CH_S models are ZWD models that only provide the ZWD part. This paper discusses the effects of each model on the total tropospheric delay. In order to compare the effects more easily, it is required to compute the ZHD with the Saastamoinen equation when we utilize the CH and CH_S models to obtain the ZTD [27], which is applied to the following statistical analysis.

In order to describe clearly the characteristics of each model, Table 1 lists the details for all models, including abbreviations, functions, types, and input parameters. In the following table, L and DoY represent longitude and Day of Year.

## 3. Data and Methods

### 3.1. Description of Radiosonde Data and IGS Data

A radiosonde is a balloon-borne platform and can directly measure meteorological parameters such as atmospheric pressure, temperature, water vapor pressure, and relative humidity at different altitudes from the ground surface to a height of over 30 km [28,29]. Using Equation (6), the ZTD over a station can be computed by using measured meteorological parameters from different heights. This study planned to utilize radiosonde data to calculate accurate ZHD and ZTD values to establish and validate each site-based model. The upper height that a radiosonde balloon can reach is limited; therefore, the ZTD over these stations would deviate slightly from the true value [27]. In order to ensure that the ZTD derived from the radiosonde data were sufficiently accurate, only radiosonde data with complete meteorological parameter profiles up to or greater than an altitude of 24 km were used in this study [5]. Moreover, we also removed those stations where the radiosonde data were discontinuous during the study period. Considering the altitude at which the radiosonde balloon reaches its limit, it is necessary to use the Saastamoinen equation to compute the delay in the neutral atmosphere above that altitude [24]. Based on the accuracy analysis of the ZHD and the ZWD obtained using radiosonde data by Chen, the theoretical accuracy of the computed ZHD and ZWD were 2.40 mm and 4.39 mm respectively when the radiosonde balloon reached a sufficient altitude (24 km or higher) [22].

**Figure 1:**
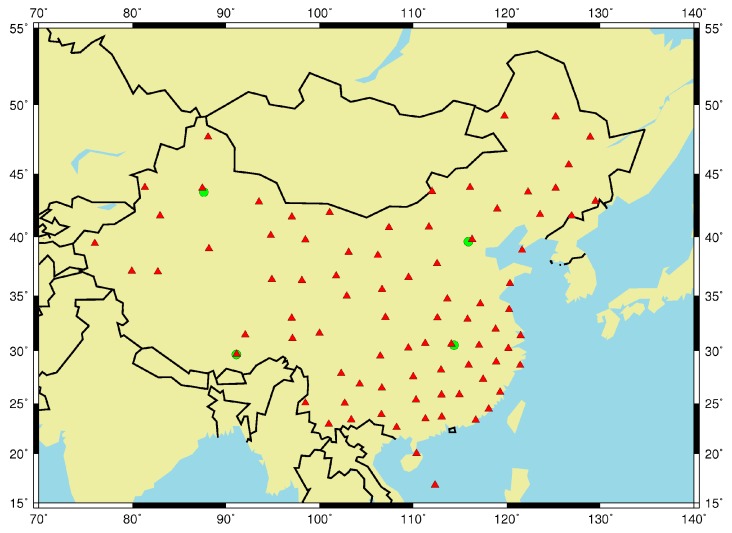
Geographic distribution of 81 radiosonde stations (red solid triangles) and 4 International Global Navigation Satellite System Service (IGS) stations (green solid circles).

In order to achieve a statistically meaningful conclusion for the model and according to the above requirements of the selected station, a large volume of radiosonde data collected from 81 stations in China over a 9-year period from 2005 to 2013 were utilized to establish and evaluate different tropospheric models. Data for 2005–2012 were used for modeling and data of 2013 was used for testing. Figure 1 shows the geographic distribution of the 81 radiosonde stations. Generally, radiosonde data are measured 1–4 times a day, but in China, the measurements occur twice a day. Therefore, the total number of radiosonde data during the period from 2005 to 2012 should be about 5840. It should be noted that the least amount of radiosonde observations for modeling was 3768, and its loss number should be 2072, so its loss rate was about 35.4%. Similarly, the least number of radiosonde data for validating was 450 with a loss rate of approximately 38%.

In addition to radiosonde data, this study used high-accuracy ZTD data provided by IGS to validate the reliability of all tropospheric models, and the RMS value and temporal resolution of the data were 1.0~5.0 mm and 5 min respectively [30]. These ZTD data are directly estimated from raw GPS range measurements employing a precise point positioning (PPP) approach [31] and using the IGS Combined Final orbit and clock product. We utilized ZTD data for 2013 collected from four Chinese IGS stations to validate all tropospheric models, and these stations are BJFS, WUHN, URUM, and LHAZ shown as green solid circles in Figure 1.

### 3.2. Model Processing

According to meteorological data from the radiosonde stations, we computed the atmospheric refractivity using Equation (2), and the ZTD value was obtained by computing the integral of atmospheric refractivity using Equation (6). We used the Saastamoinen equation to compute the ZTD value, we use the Saastamoinen equation to compute the delay above the height that a radiosonde balloon can reach. Then, we calculated the ZHD with Equation (7) and obtained the ZWD by subtracting the ZHD from the ZTD. Next, we used the multiple-year ZTD and ZWD time series data from January 2005 to December 2012 to determine the coefficients of the SAAS_S and CH_S models using a least squares estimation method. Subsequently, we validated all models with radiosonde data from 2013. In addition, to analyze the influence on the results of the single-site tropospheric models using data from different periods, we established these two models using data from 2005–2008, 2009–2010, 2009–2012, 2011–2012, and 2005–2012. Then we evaluated the accuracy of these two models using the data for 2013. In addition, this study utilized ZTD data for 2013 from IGS stations in China to further test all tropospheric models. The detailed computational formulas for the fitting errors and the model accuracies are shown in the following. Equation (15) is utilized to calculate the model error, and Equations (16) and (17) are the formulas for determining the model accuracy.

(15) $$\sigma =\sqrt{\frac{V^{T}V}{N}}$$

(16) $$bias=\frac{\sum_{i=1}^{M}\left(\mathrm{ZT}D_{i}^{j}-\mathrm{ZT}D_{i}^{\mathrm{mod}el}\right)}{M}\;j=1\;or\;2$$

(17) $$\mathrm{RMS}=\sqrt{\frac{\sum_{i=1}^{M}{\left(\mathrm{ZT}D_{i}^{j}-\mathrm{ZT}D_{i}^{\mathrm{mod}el}\right)}^{2}}{M}}\;j=1\;or\;2$$

where σ stands for fitted model error, V is the vector of the fitted residuals, and N stands for the total number of data involved in the statistics. The accuracy of the model includes the bias and Root Mean Square, which are respectively denoted by bias and RMS in Equations (16) and (17). Here, $\mathrm{ZT}D_{i}^{j}$ stands for the actual ZTD values from the radiosonde data (j = 1) or the IGS data (j = 2), $\mathrm{ZT}D_{i}^{\mathrm{mod}el}$ are the ZTD values computed by the tropospheric models, and i and M are the ith value and the total number of data involved in the comparison respectively.

**Figure 2:**
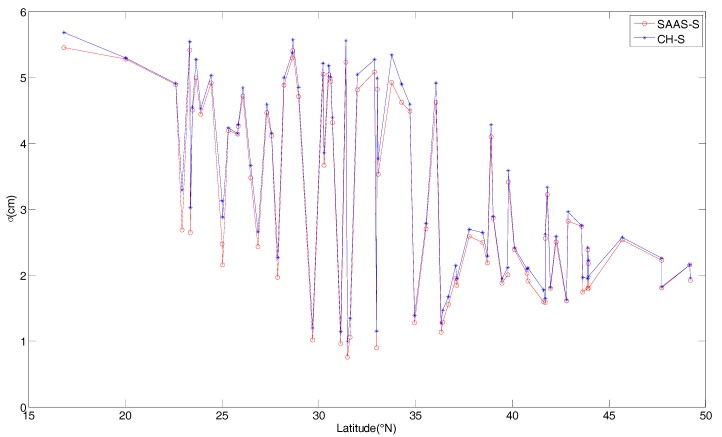
The variations of σ for the two single-site models in different regions.

In order to analyze the fitting effect of the model coefficients, Figure 2 provides the variations of the fitting error for the two single-site tropospheric models in different latitude regions. The σ of the two single-site models basically ranges from 0.75 cm to 5.68 cm, and the σ values of the SAAS_S model are slightly smaller than those of the CH_S model. With the increase in latitude, the σ values decrease, and the fitting errors of the single-site models gradually decrease as well.

## 4. Results

According to the above methods, all tropospheric models are validated with data from 81 radiosonde stations and 4 IGS stations. Detailed results are given in the following.

### 4.1. Validation with Radiosonde Data

Firstly, the validation results of those tropospheric models with the radiosonde data are presented. Table 2 shows the mean accuracy for the SAAS, SAAS_S, CH, CH_S, GPT2w, and GPT2w_S models at all radiosonde stations.

Table 2 shows that the two single site models, SAAS_S and CH_S, obviously achieve a higher accuracy than their original global models (SAAS and CH), respectively. The mean bias values of the SAAS_S and CH_S models are 0.19 cm and −0.05 cm, respectively, and their mean RMS values are 3.19 cm and 3.38 cm, respectively. The mean bias values of these models are closer to zero than those of the SAAS and CH models, and the improvements in the mean RMS value are 0.43 cm and 0.60 cm respectively, which indicates that the development of single-site tropospheric models is advantageous. In addition, the mean bias and RMS values of the GPT2w_S model show an obvious improvement compared to the GPT2w model, which indicates that using observed meteorological parameters leads to better ZTD prediction results. From the statistical result of Table 2, we find that the accuracy of the SAAS_S model is the highest, followed by the CH_S model; the accuracy of the GPT2w_S model is slightly superior to the SAAS model; and the accuracy of the GPT2w models is the worst.

**Figure 3:**
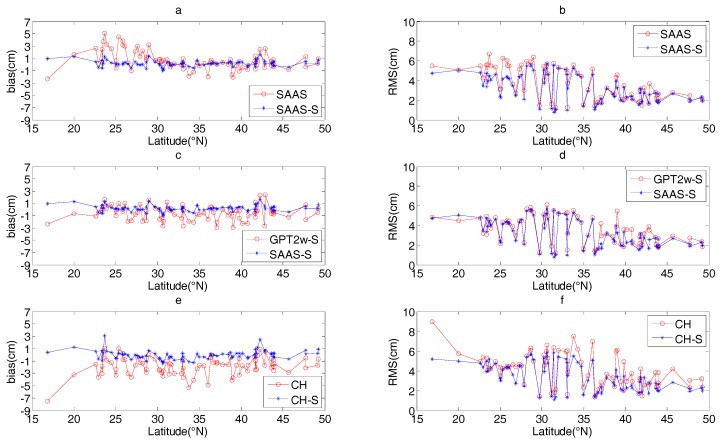
Comparison of the accuracy of the three groups of tropospheric models in different regions. The detailed models of each subgraph are as follows: (**a**) and (**b**) include the SAAS and SAAS_S models; (**c**) and (**d**) include the GPT2w_S and SAAS_S models; (**e**) and (**f**) include the CH and CH_S models.

In order to illustrate the performance of these tropospheric models in the different regions, Figure 3 shows the bias (left column) and RMS (right column) values at each station, with stations aligned with respect to latitudes. Figure 3a,b show the a detailed comparison between SAAS and SAAS_S, similar results for CH and CH_S is found in Figure 3e,f. In addition, we also present the comparison between SAAS_S and GPT2w_S models in Figure 3c,d, which are a single site model built in this study and a gridded single site model.

As is evident from Figure 3, in most regions, the bias and RMS values of the SAAS_S and CH_S models are smaller than those of the SAAS and CH models, which shows that the single-site tropospheric models developed in this study provide a better accuracy than the corresponding global models. Figure 3a,b show that in most sites at latitudes between 15°N~30°N, the absolute value of the biases of the SAAS_S model are about 0.55~4.51 cm smaller than those of the SAAS model, and the RMS values are about 0.51~2.12 cm smaller than those of the SAAS model. This indicates that in many cases the coefficients of the SAAS model can not represent the real tropospheric environment, while the coefficients of the SAAS_S model are more accurate because they are based on local data. Therefore, in the lower latitude regions of China, the more accurate model SAAS_S should be employed by GNSS users. In the middle and high latitude regions, the accuracies of the SAAS_S and SAAS models are similar, but at some sites (for example, stations 51777 and 54292), the RMS values of the SAAS_S model is obviously higher than those of the SAAS model. As is evident from Figure 3c,d, it is discovered that the RMS values of the SAAS_S model are about 0.50~2.20 cm smaller than those of the GPT2w_S model at 15 sites, and at the other sites the RMS values of the two models are basically identical. The absolute values of the biases for the SAAS_S model are about 0.50~2.90 cm smaller than those of the GPT2w_S model at 47 sites. In order to validate whether the better accuracy of SAAS_S are due to using recent period of data to establish the model, we conducted a further analysis for the sites where the absolute value of the biases of the SAAS_S model are smaller than those of the GPT2w_S model. We found that the absolute value of the biases of the SAAS_S model developed using data for periods (2005–2008 and 2009–2010) are basically smaller than those of the GPT2w_S model. This phenomenon demonstrates that the coefficients of the SAAS_S model are more in tune with the local climatic conditions. In addition, the SAAS_S model can also provide more precise ZTD values for partial regions in China, while the coefficients of the GPT2w_S model are less accurate there. Furthermore, we find that the accuracy of the SAAS_S model is very close to that of the CH_S model at most sites, but the accuracy of the SAAS_S model is superior to that of the CH_S model at individual sites, which shows that developing models based on the surface pressure, temperature, and vapor pressure is reliable. As is shown in Figure 3, the bias values for the SAAS_S, CH_S, and GPT2w_S models range from −1.03~1.65 cm, −1.34~4.05 cm, and −2.97~2.39 cm respectively, and the RMS values for the SAAS_S, CH_S, and GPT2w_S models range from 0.77~5.70 cm, 1.11~5.82 cm, and 1.06~6.09 cm respectively. These values show that the SAAS_S model has the highest accuracy among the three models, and that the CH_S model is slightly superior to the GPT2w_S model. Also, we discovered that the RMS values for the five models decrease with an increase in latitude, which indicates that the accuracy of the five models increases with an increase in latitude, very likely due to the increased humidity in low latitude regions. This finding is in agreement with the results reported by Li et al. and Yao et al. [15,16].

Penna et al. found that a 1 cm error in the ZTD can result in a height error of about 3.6 cm for the 5 degree elevation angle cut-off case with elevation angle weighting, and of about 2.6 cm for the 15 degree elevation angle cut-off case with elevation angle weighting [13]. Since the mean RMS value of the SAAS_S model is 0.43 cm smaller than that of the SAAS model, the mean positioning errors in vertical components can be reduced by about 1.12~1.55 cm when the SAAS_S model is applied to the positioning of GNSS instead of the SAAS model. At these sites, in low latitude regions where the RMS values of the SAAS_S model are about 0.51~2.12 cm smaller than those of the SAAS model, the positioning errors in vertical components can be reduced by about 1.33~7.63 cm when the SAAS_S model is applied to the positioning of GNSS instead of the SAAS model. These phenomena show that the mean accuracy of vertical direction in China region can be improved by 1.12~1.55 cm and the accuracy of vertical direction in low latitude areas can be improved by 1.33~7.63 cm when the SAAS_S model is replaced by the SAAS model in the positioning of GNSS.

**Figure 4:**
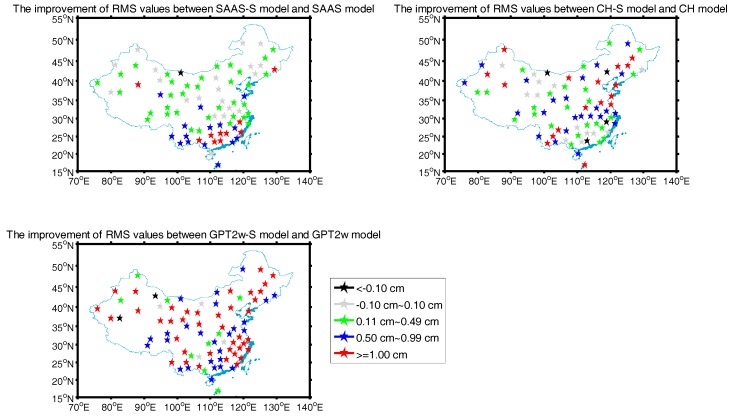
The improvement in the RMS values for the three pairs of tropospheric models.

At most of the radiosonde stations, the RMS values of the corresponding models show a certain improvement compared to the SAAS, CH, and GPT2w models. In order to distinguish the detailed improvement in the RMS for the three pairs of models, we use black, gray, green, blue, and red to represent the five different types of improvement in the RMS value in Figure 4. Here, black represents that the RMS values of the new models are inferior to those of the existing models, and gray indicates a difference in the RMS values of −0.10~0.10 cm; we assume that the errors for the two models are identical when their differences are located in this range. While, green indicates an improvement in the RMS value of 0.11~0.49 cm, blue shows an improvement in the RMS value of 0.50~0.99 cm, and red shows an improvement of greater than or equal to 1 cm. The detailed results are given in Figure 4.

The results confirm that at 60 sites the RMS values of the SAAS_S model show a certain improvement over the SAAS model, and at 25 sites, the improvement in the RMS values is greater than or equal to 0.50 cm. We discovered that the improvement of the RMS values between the SAAS_S model and the SAAS model is clearly evident at latitudes in the range of 15°~30°N. Additionally, at 64 sites, the RMS values of the CH_S model exhibit a certain improvement compared to the CH model, and at 40 sites, the improvement of the RMS values between the CH_S model and the CH model is greater than or equal to 0.50 cm. These phenomena show that the improvement of the RMS values between the single-site models and the global models is considerable. In addition, at 75 sites, the RMS values of the GPT2w_S model show a certain improvement compared to the GPT2w model, and at 66 sites, the improvement of the RMS values between the GPT2w_S model and the GPT2w model is greater than or equal to 0.50 cm. This indicates that for most regions in China, the improvement of the RMS value between the GPT2w model and the GPT2w_S model is obvious.

On the other hand, we also analyzed the stations where the SAAS_S, CH_S, and GPT2w_S models show no better performance than their corresponding models. We found that only at station 52267, the RMS value of the SAAS_S model is 0.25 cm larger than that of the SAAS model. This can be due to erroneous radiosonde data used for calculating the reference ZTD. The RMS value of the CH_S model is 0.14~0.94 cm larger than that of the CH model at 4 radiosonde stations, which shows that it is not effective to only consider the surface temperature and vapor pressure to develop a single-site model; however, it is reliable to take into consideration the surface pressure, temperature, and vapor pressure. In addition, the RMS value of the GPT2w_S model is greater than that of the GPT2w model at two radiosonde stations with values of 1.07 cm and 1.42 cm respectively.

In order to discuss the seasonal variations of these tropospheric model errors, Table 3 shows the statistics of these models for spring, summer, autumn, and winter. Spring consists of March, April, and May, summer consists of June, July, and August, autumn consists of September, October, and November, and winter consists of December, January, and February. Table 3 indicates that the deviations of all tropospheric models are basically identical for the different seasons, with the largest error in summer, the smallest in winter, and a slightly larger error for autumn than for spring. The reason is the greater temporal and spatial variability of the atmospheric water vapor distribution during summer and autumn compared to spring and winter, which is a common challenge for empirical tropospheric models [21]. In addition, compared to the SAAS, CH, and GPT2w models, the RMS values of their corresponding single-site models show a certain improvement in all four seasons. The improvement of the RMS value for the SAAS_S model is larger in the summer with a value of 0.74 cm. The improvement of the RMS value for the CH_S model is more firm in autumn with a value of 0.92 cm. The improvement of the RMS value for the GPT2w_S model is strongest in the summer and autumn, and the values are 1.08 cm and 1.09 cm, respectively. This indicates that the improvement of the RMS values for the three pairs of models is obvious in the summer and autumn.

**Figure 5:**
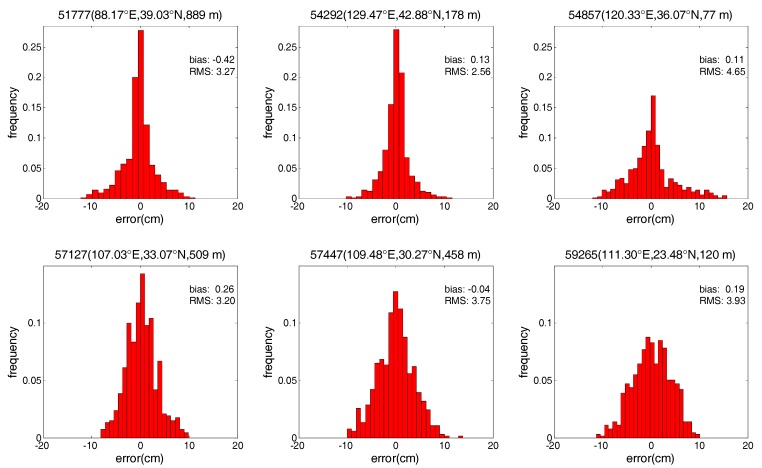
Frequency histograms of error distribution for the SAAS_S model at six radiosonde stations.

**Figure 6:**
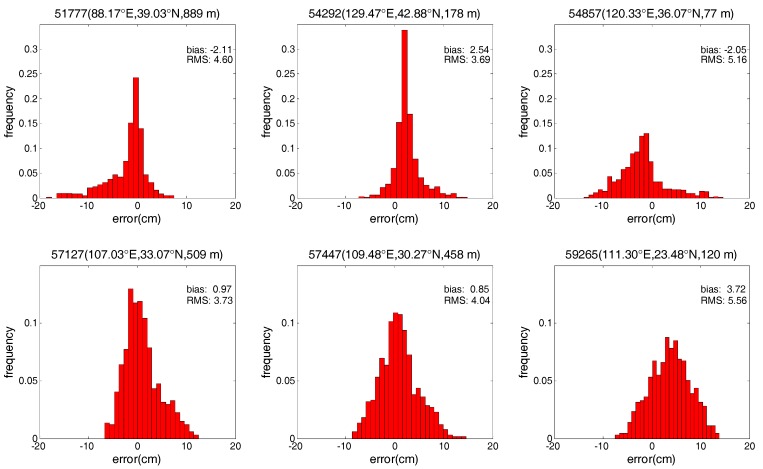
Frequency histograms of error distribution for the SAAS model at six radiosonde stations.

In addition, to show more detailed error characteristics for the SAAS_S and SAAS models, this study also analyzes the changes in the error for the two models at six exemplary radiosonde stations. In order to show the detailed improvement in accuracy between the SAAS_S model and the SAAS model, we select the six stations where the RMS values of the single-site models are smaller than those of the global models. Similar results are obtained at other stations. Figure 5 and Figure 6 depict the frequency histograms of the error distribution for the three models in 2013 at six radiosonde stations with station numbers of 51777, 54292, 54857, 57127, 57447, and 59265. Stations 57127 and 57447 are located in central China, 54292 and 59265 are located in the north and south of China, and 51777 and 54857 are located in the west and east of China respectively.

As can be seen from Figure 5 and Figure 6, the SAAS_S model has no obvious systematic errors at these six stations. In addition, most of the errors of the SAAS_S model are within ±9 cm and generally follow the standard normal distribution. In contrast, most of the errors of the SAAS model are within −15~12 cm, and most of the errors of are less centralized than those of the SAAS_S model. These phenomena show that at most radiosonde stations the accuracy of the single-site models is higher than that of the global models. Moreover the residuals of the SAAS_S model are closer to a normal distribution compared to those of the SAAS model.

**Figure 7:**
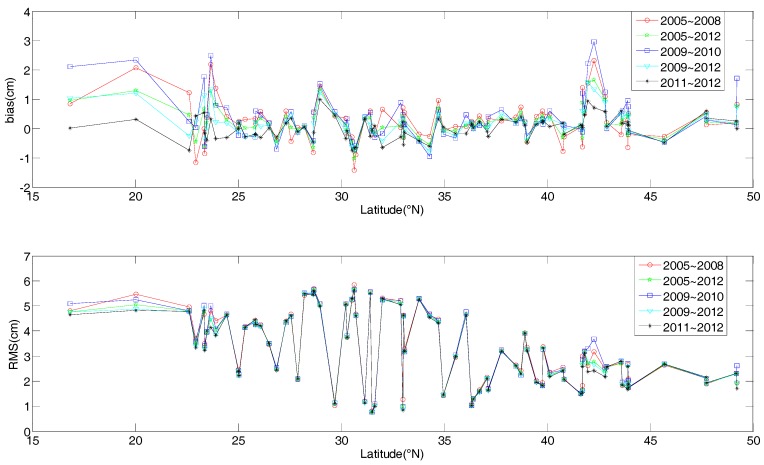
Comparison of the accuracy for the SAAS_S model based on data from different periods in different regions.

To analyze the influence of using radiosonde data from different periods on the SAAS_S and CH_S models, the two models are modeled with data from 2005 to 2008, from 2009 to 2010, from 2009 to 2012, from 2011 to 2012, and from 2005 to 2012 respectively, and are validated with radiosonde data from 2013. Then we compared the individual quality of the two established models with data for different periods. The statistical characteristics of the specific comparison for the SAAS_S model is shown in Figure 7.

As can be seen from Figure 7, the quality of the models developed using radiosonde data from 2005 to 2008 and from 2009 to 2010 are obviously lower than those of the model developed with data from 2009 to 2012, from 2011 to 2012, and from 2005 to 2012. This shows that models based on data closer to the time period of validation data result in slightly better modeling performance, which is due to higher correlation with actual atmospheric conditions for nearby years. Therefore, we can use the recent data to update the coefficients of the single-site models. In addition, we find that the accuracies of the established model with data from 2011 to 2012 and from 2009 to 2012 are similar to the accuracy of the established model with data from 2005 to 2012. When only short period data (for example 2 or 4 years) is available, establishing a single-site model based on those data can also achieve a satisfactory accuracy.

### 4.2. Validation with GNSS-ZTD Data

**Figure 8:**
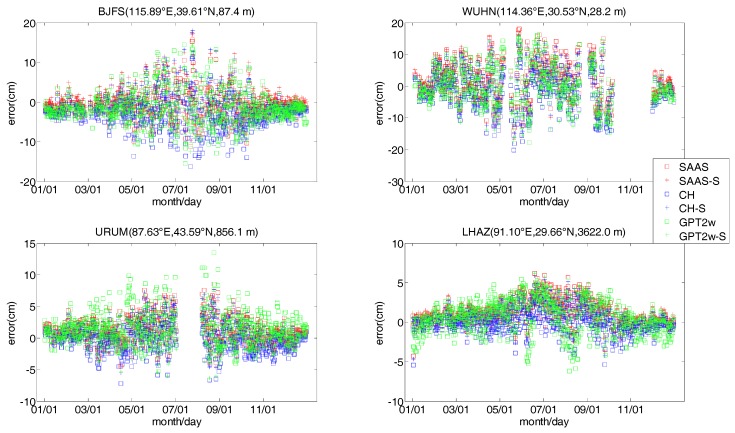
Changes in the error for the tropospheric models at the IGS stations.

To validate the reliability of all tropospheric models further, we downloaded ZTD data for 2013 from four IGS stations to analyze the models in this section. We selected four radiosonde stations located close to the IGS stations. The horizontal distances between the IGS station and the radiosonde stations are 5.31 km for LHAZ, 29.94 km for WUHN, 36.83 km for URUM, and 39.53 km for BJFS. Generally, the tropospheric errors in the propagation path are believed to change slightly over a range of tens of kilometers [32]. Therefore, it is feasible to validate the single-site tropospheric models developed with radiosonde data from 2005 to 2012 using ZTDs from nearby IGS stations. Meanwhile, we also account for the difference in sensor heights between the IGS and the radiosonde station in the validation process. Figure 8 shows the time series of all model errors and Table 4 shows the bias and RMS errors for all models at each site. Because radiosonde data were missing for July and August at the URUM station and a similar situation also occurred at WUHN, the second and third subgraphs in Figure 8 have some gaps.

As can be seen from Figure 8, the errors of each model at BJFS, WUHN, URUM, and LHAZ have apparent seasonal changes, and the absolute values and the variation of these errors are larger in summer, but smaller in the winter. About 90% of the errors for all models are within ±10 cm at BJFS, and are within ±5 cm at URUM and LHAZ, but the errors of the SAAS, CH, and GPT2w models fluctuate greatly. Additionally, about 80% of the errors for all models are within ±10 cm at WUHN, and the errors fluctuate much more in summer, which is likely related to continuously high temperatures and air humidity in the summer in Wuhan.

As is evident from Table 4, compared to the SAAS models, the SAAS_S model shows a certain improvement in the bias and RMS value at four IGS stations. In addition, the RMS value at URUM and LHAZ are improved by 0.44 cm and 0.54 cm, respectively. The accuracy of the SAAS_S model is very close to that of the GPT2w_S model at WUHN and URUM, but the accuracy of the SAAS_S model is clearly higher than that of the GPT2w_S model at BIJF and LHAZ. Compared to the CH model, the bias and RMS values of the CH_S model show an improvement at BJFS and WUHN, and the improvement is larger at BJFS with a value of 1.43 cm. However, the bias and RMS values of the CH_S model are bigger than those of the CH model at URUM and LHAZ, which shows that it is defective to only consider the surface temperature and vapor pressure to develop a single-site model. Additionally, the GPT2w_S model also shows an improvement in the RMS value compared to the GPT2w model at four stations, and the improvement are greater at BJFS and URUM with values of 1.03 cm and 1.31 cm respectively. However, the absolute value of the bias for the GPT2w model is lower than that of the GPT2w_S model at BJFS, WUHN, and LHAZ. In summary, at the above four IGS stations, most of the RMS values for the SAAS_S, CH_S, and GPT2w_S models show an improvement compared to the SAAS, CH, and GPT2w models, and the accuracy of the SAAS_S model is the highest among the four single-site models.

### 4.3. Characteristics of the SAAS_S Model Coefficients

**Figure 9:**
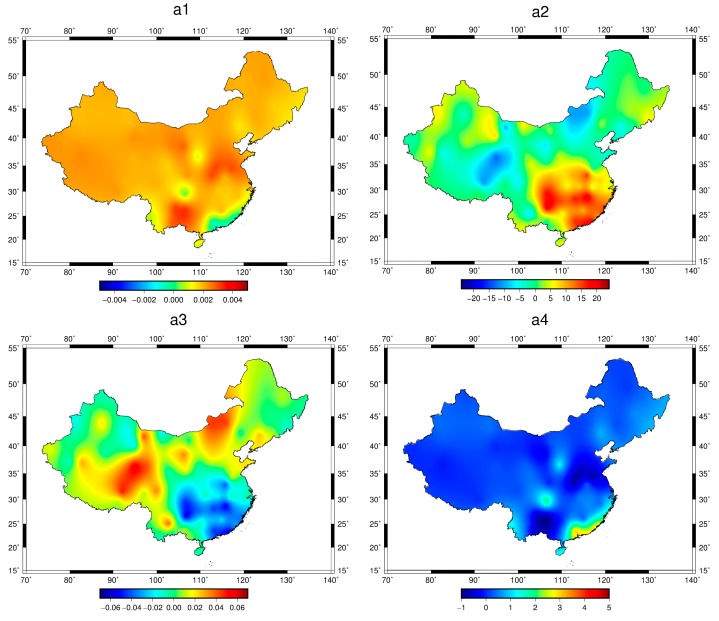
The distribution of the SAAS_S model coefficients. The four subfigures (**a1**–**a4**) stand for the spatial distribution of the four coefficients (a1–4), respectively.

As can be shown from Equation (14), the SAAS_S model approximates the Saastamoinen model, but it has an additional constant term. In this section, we describe a detailed analysis of the spatial distribution of the SAAS_S model coefficients in China. With the 81 pairs of coefficients for the SAAS_S model shown in Figure 1, we used the Surfer software to depict the changes in distribution for each model coefficient. The Surfer software can be found at http://www.goldensoftware.com/products/surfer. The four subgraphs in Figure 9 show the spatial distribution of the Coefficients (a1–4) based on Equation (14).

Based on Equation (14), (a1) is related to hydrostatic delay, while (a2) and (a3) are related to wet delay. The detailed variations in Figure 9 show that (a1) and (a4) have small changes at different geographic locations, while (a2) and (a3) change considerably more. Specifically, the value of (a1) is closer to the coefficient term of the SAAS model associated with the hydrostatic delay, and its spatial variation is very small, which indicates that the coefficients related to the hydrostatic delay are basically identical in different areas. Similarly, the spatial variation of (a4) is also very small. Coefficient (a1,4) are highly negatively correlated, the variation of (a1) is small, and (a4) is a kind of regional compensation. The values of (a2) and (a3) change considerably, which shows that a wet delay has obvious regional characteristics and its difference is relatively large for the different regions. However, the value of (a2) increases with a decrease in latitude, and the value of (a2) is larger in the southeast region than in the other regions. By contrast, the change of (a3) is opposite to (a2), showing that the value of (a3) is smaller in the southeast region than in the other regions.

## 5. Conclusions

Based on the formula of the Saastamoinen and Callahan models, this study has developed two single-site tropospheric models (namely: SAAS_S and CH_S models) for the China region using radiosonde data from 2005 to 2012. We validated the two new single-site tropospheric models using radiosonde data for 2013 and ZTD data from four IGS stations, and compared them to the results of the SAAS, CH, GPT2w, and GPT2w_S models. The conclusions of the study are as follows:

The mean accuracy of the SAAS_S model (bias: 0.19 cm, RMS: 3.18 cm) in China is superior to that of the SAAS, CH, CH_S, and GPT2w_S models. In most regions of China, the accuracies of the SAAS_S, CH_S, GPT2w_S models show a certain improvement compared to their source models. The SAAS_S model exhibits a clear improvement in the accuracy over the Saastamoinen model in low latitude regions, which shows that in many cases the coefficients of the SAAS model can not represent the real tropospheric environment. In addition, the RMS values of the SAAS_S, CH_S, and GPT2w_S models show a certain improvement compared to their source models in all four seasons, and the improvement is very obvious in the summer and autumn. When the SAAS_S model is replaced by the SAAS model in the positioning of GNSS, the mean accuracy of vertical direction in the China region can be improved by 1.12~1.55 cm and the accuracy of vertical direction in low latitude areas can be improved by 1.33~7.63 cm.

The residuals of the SAAS_S model are closer to a normal distribution compared to those of the SAAS model.

By using radiosonde data spanning different time intervals to build the single-site tropospheric models, we find that models based on data closer to the time period of validation data result in slightly better modeling performance, which is due to higher correlation with actual atmospheric conditions for nearby years. Besides, using the short period of the most recent data (for example, 2 years) to build the model can also achieve satisfactory accuracy.

The SAAS_S model coefficients (a1,4) exhibit small spatial variation, but (a2) and (a3) both change considerably for different geographic locations. Moreover, (a1) has a negative correlation with (a4), which is similar to the relationship between (a2) and (a3).

In summary, the SAAS_S and CH_S models in this study have a good accuracy, and their calculations are simple and fast. In addition, the SAAS_S model has a higher accuracy than the CH_S model, and we recommend the model to those users who can obtain the surface measured meteorological parameters (especially in low latitude areas).

## Figures and Tables

**Table 1:** Abbreviation, equations, types, and input parameters for tropospheric models.

Model	Label	Equation	Type	Input Parameters
Saastamoinen	SAAS	(8), (9)	Global	e, P, T, ϕ
Saastamoinen_S	SAAS_S	(14)	Single-Site	e, P, T, ϕ
Callahan	CH	(7), (13)	Global	e, T
Callahan_S	CH_S	(7), (13)	Single-Site	e, T
GPT2w	GPT2w	(7), (10), (11)	Single-Site	L, ϕ, h, D
GPT2w_Surface	GPT2w_S	(7), (10), (11)	Single-Site	ϕ, h, e, P, T

**Table 2:** Statistical results of the mean accuracy using radiosonde data for 2013.

Model	Bias/cm	RMS/cm
SAAS	0.62	3.62
SAAS_S	0.19	3.19
CH	−1.89	3.98
CH_S	−0.05	3.38
GPT2w	−1.31	4.40
GPT2w_S	−0.59	3.46

**Table 3:** Statistical data for various tropospheric models in different seasons (unit: cm).

Model	Spring		Summer		Autumn		Winter		Mean	
	Bias	RMS	Bias	RMS	Bias	RMS	Bias	RMS	Bias	RMS
SAAS	0.43	3.45	2.11	5.02	0.01	3.71	−0.05	2.29	0.62	3.62
SAAS_S	−0.01	3.06	1.07	4.28	−0.43	3.39	0.13	2.04	0.19	3.19
CH	−1.84	3.88	−1.68	4.75	−2.55	4.49	−1.48	2.78	−1.89	3.98
CH_S	−0.29	3.19	1.49	4.49	−0.73	3.57	−0.65	2.26	−0.05	3.38
GPT2w	−1.32	4.23	−1.00	5.65	−1.76	4.77	−1.16	2.96	−1.31	4.40
GPT2w_S	−0.68	3.37	−0.36	4.57	−0.88	3.68	−0.48	2.22	−0.59	3.46

**Table 4:** Statistical results for all tropospheric models computed by using data from 2013 for four IGS stations (unit: cm).

Model	BJFS		WUHN		URUM		LHAZ		Mean	
	Bias	RMS	Bias	RMS	Bias	RMS	Bias	RMS	Bias	RMS
SAAS	−1.19	3.54	1.98	6.39	1.30	2.34	1.35	2.07	−0.86	3.59
SAAS_S	0.57	3.37	1.21	6.16	0.60	1.90	0.95	1.53	0.83	3.24
CH	−3.55	5.01	−1.82	6.33	−0.29	1.97	−0.01	1.35	−1.42	3.67
CH_S	−0.33	3.58	0.49	6.07	0.77	2.12	0.87	1.74	0.45	3.38
GPT2w	−1.39	4.65	0.71	6.20	1.79	3.16	0.29	2.13	0.35	4.04
GPT2w_S	−1.52	3.62	−1.13	6.11	0.35	1.85	1.49	2.12	−0.20	3.43
